# Monotonous Diet

**Published:** 1890-02-01

**Authors:** 


### DIETETICS.

### MONOTONOUS DIET.

"The Diet of Monotony" is the title of an article by Mr. W. Armstrong Willis in the last number (January) of the *Provincial Medical Journal*. It is a matter of surprise to the writer that middle-class English cooking should remain so stationary and unadvanced in character. The poor dyspeptic man must often sigh, and groaningly wish that some of the skill and thought expended by women on other matters were diverted to producing more variety and daintiness in the daily food. Sir Henry Thompson says that on questioning the middle-class Englishman as to the nature of his food the all but universal answer is, "My living is plain, always roast and boiled." But as regards roasting, we now rarely get roast meat, for it is usually baked in a cooking range. The author agrees with Sir Henry Thompson that braising is a superior method of cooking meat to baking it in an oven. Nowhere do we get better vegetables than in England, but nowhere are they so unskilfully and rudely cooked. Treated judiciously they should form an important part of our dinner. But it is often difficult for a man of sedentary habits and delicate appetite to get a suitable meal for want of appetising light vegetable dishes. The question of salads is also an important one. "A salad in England generally resembles the meal a child gives its pet rabbit." It never occurs to the cook how delicious even a simple lettuce can be made by serving it crisp and fresh, each leaf lightly brushed over with Lucca oil, a little salt and vinegar being added just before serving." Anglo-Saxons who declare they never touch oil, and hate it in every shape and form, may be seen in hundreds consuming such a mild salad with avidity at any *table d'hote* in Paris." The same neglect with which salads have been treated extends to soups, fish, stews, and omelettes. The whole treatment of fish and its wilful neglect is a theme in itself for a volume. The author, however, has something in praise of Cornwall cooking, especially of the Cornwall pasty, whether made of vegetables, of meat, or of fish, or of a combination of two of such materials. It is traditional that the evil one never enters Cornwall for fear he should be caught and put in a pasty. Mr. Willis says if he did, the Cornwall housewife would make something palateable and tasty of him! But in the South of England generally there exists scarcely any more power of cooking than among the natives of Terra del Fuego. Hence the labourers and their families are often victims of dyspepsia. The fire is only used to boil the kettle, and to brew the harsh astringent drink miscalled tea. They live on hunches of bread with American cheese, and for a treat they substitute a debauch of fat bacon, washed down with floods of bad beer, or cider as harsh as acetic acid. Mr. Willis thinks they do these things better in France. The French peasant has always a "stock-pot" going, which is the basis of soup. Mr. Willis further advises to turn at first hand to some of the manuals of middle-class French cookery. "Variety," says the author, "is the salt of life," notwithstanding that physicians, scientists, and philanthropists are dealing with the food question from elevated and intellectual heights. Now there is a good deal of truth in what Mr. Willis advances, but we think that he rather over-rides a hobby. One of the numerous sayings attributed to Sydney Smith is that "a man who eats a plain joint is little better than a cannibal." We believe, however, that a plain, well-cooked joint is more nutritious and digestible than most made dishes. Garth said, "No cook with art increased physicians' fees, nor served up death with soup and fricassees." We imagine Mr. Willis would not prefer the dinner of herbs to the variety of an ordinary feast, with its Julienne soup, filleted sole *à la maître d'hotel*, Dartmoor mutton, a brace of pheasants, curry as attempted in England, sweet preparations of a gelatinous and farinaceous order, Stilton cheese, biscuits, and fossil, crystalised fruit, etc., etc. But we undertake to say that the person who habitually lives on plain roast and boiled will have a better digestion than the one who takes a *table d'hote* repast daily. At the same time we are fully sensible that the British matrons as a class do not cook well, and do not make the most of their material.

